# Microwaved schiff base dialdehyde cellulose-chitosan hydrogels for sustained drug release with DFT calculations

**DOI:** 10.1186/s13065-025-01469-3

**Published:** 2025-05-02

**Authors:** Hebat-Allah S. Tohamy

**Affiliations:** Cellulose and Paper Department, National Research Centre, 33 El Bohouth Str. Dokki, P.O. 12622, Giza, Egypt

**Keywords:** Dialdehyde cellulose, Chitosan, Schiff base hydrogels, Recycling, 4-Amino acetophenone, Drug loading, DFT

## Abstract

Dialdehyde cellulose (DAC) prepared from sugarcane bagasse (SC) by an eco-friendly, fast and low-cost microwave method was used for loading and sustained release of 4-aminoacetophenone (4-AAP). DAC was reacted with chitosan (Ch) and 4-AAP via a Schiff base reaction. FTIR analysis confirmed successful Schiff base formation between DAC and Ch, evidenced by the disappearance of the DAC aldehyde peak at 1716 cm⁻^1^ and the appearance of the imine peak at 1631 cm⁻^1^, as well as strong hydrogen bonding with incorporated 4-AAP, indicated by a shift in the O–H stretch from 3336 cm⁻^1^ to 3330 cm⁻^1^.Swelling studies showed increased water absorption with higher 4-AAP content, with 4-AAP@DAC/Ch2 demonstrating pseudo-second-order kinetics and non-Fickian diffusion. The DFT calculations revealed that the 4-AAP@DAC/Ch hydrogel exhibited enhanced stability and reactivity. A significantly reduced HOMO–LUMO energy gap, coupled with negative Pi values, indicated strong interactions between DAC, chitosan, and 4-AAP. The high adsorption energy further supported the observed slow drug release, validating the experimental findings.

**Graphical Abstract:**

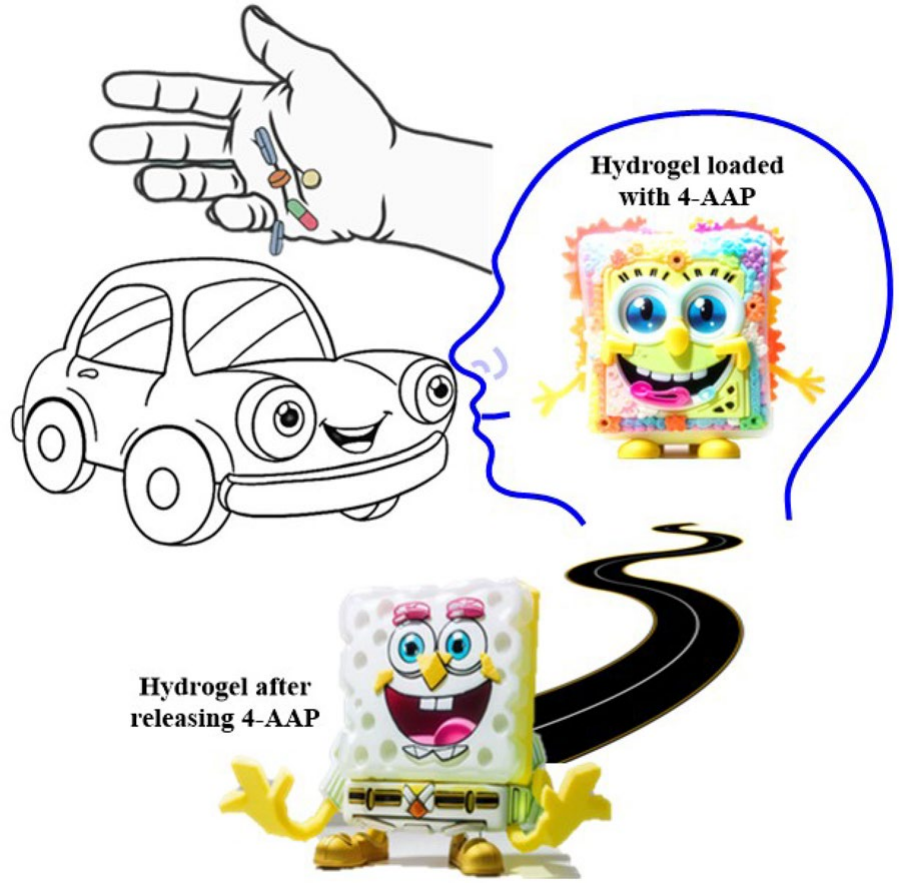

## Introduction

Sustained-release drug delivery systems offer precise and controlled drug distribution, maintaining target blood levels and optimizing therapeutic effects. This approach can enhance both efficacy and patient convenience by tailoring drug release to specific requirements or directing it to particular locations [1–3]. Matrix systems can be modified to accommodate various medications and treatment needs [4–6]. By adjusting parameters such as drug loading and polymer type, we can design matrix systems with desired release profiles, making them valuable tools for improving drug delivery and patient outcomes [3, 7]. Matrix approaches are widely used to control drug release from pharmaceutical dosage forms. Matrix systems, designed to control the release of drugs from pharmaceutical dosage forms, are commonly used for sustained or delayed drug delivery. Hydrophilic polymer matrices are particularly favored in oral drug delivery due to their versatility, cost-effectiveness, and regulatory acceptance. Drug release from these matrices is a complex process involving the interplay of erosion, diffusion, and dissolution [3, 8]. Natural polymers play a crucial role in the development of innovative controlled-release dosage for human healthcare [9–13]. Their application has significantly expanded in recent years, and they will continue to be essential in creating new formulations for controlled-release drug delivery. Compared to synthetic polymers, which carry a substantially higher risk profile, natural polymers offer biocompatibility and biodegradability. In environmental applications, they serve as effective biosorbents for heavy metal removal and in creating biodegradable packaging to combat plastic pollution [14, 15]. Moreover, their role in developing sustainable composites and films for diverse industrial applications further underscores their broad applicability [16–18]. Prominent examples of natural polymers used in drug delivery include chitosan, alginate, gelatin, cellulose derivatives (like hydroxypropyl methylcellulose or HPMC), hyaluronic acid, and starch [3, 10, 19]. Chitosan, extracted from the exoskeletons of crustaceans, presents beneficial mucoadhesive qualities and the capacity to improve drug absorption [20]. Nevertheless, its solubility is influenced by pH, and variations between production batches can arise [21]. Alginate, sourced from marine algae, creates biologically compatible and readily formed gel structures, suitable for drug encapsulation. However, regulating its breakdown speed can be problematic [22]. Gelatin, a protein from collagen, offers excellent biological compatibility and degradability, but its physical strength and temperature stability are limited [23]. Cellulose-based compounds, such as HPMC, are widely favored for their adaptability and ability to modulate drug release through altering substitution levels. However, the risk of microbial contamination during manufacturing necessitates stringent controls [24, 25]. Hyaluronic acid, a glycosaminoglycan, demonstrates high biological compatibility and inherent targeting potential, though it may be relatively costly [26, 27]. Starch, a polysaccharide, is abundant and biodegradable, but achieving precise control over its physical properties and breakdown rate can require modifications [28]. 4-AAP has a rich and lengthy history that spans centuries in the field of medication development. Its involvement in chloramphenicol, a commonly used antibiotic that is still being made today, is one excellent example [29]. Sustained release of 4-AAP is difficult due to its high solubility. 4-AAP demonstrates solubility in both polar (like ethanol and hot water) and less polar (like ether) solvents [30]. For a sustained release, the substance must be encased in a carrier.

As a readily available and low-cost resource, sugarcane bagasse (SC) provides a viable alternative to conventional cellulose sources. Furthermore, its fibrous structure and high cellulose composition make it particularly suitable for the production of cellulose. By leveraging this renewable resource, this research aims to explore the feasibility and benefits of utilizing sugarcane bagasse in the creation of dialdehyde cellulose, thereby contributing to the development of eco-friendly materials and reducing reliance on traditional, less sustainable sources [31, 32]. The most common renewable biopolymer is cellulose, which is a linear homopolymer of D-anhydroglucopyranose units (AGUs) connected by β−1,4-AGU inks [33–37]. By using chemical techniques including etherification, halogenation, esterification, and oxidation, a wide range of cellulose derivatives can be created [31, 38–41]. By carefully breaking the C_2_-C_3_ bond in each cellulose AGU unit, this ingenious technique produced paired aldehyde groups on the molecule. Dialdehydes, characterized by two aldehyde functional groups (-CHO), are inherently unstable compounds [42, 43]. This instability stems from several factors, including their susceptibility to oxidation, polymerization, and self-condensation reactions [44]. The presence of two electron-withdrawing aldehyde groups increases the reactivity of the molecule, making it prone to atmospheric oxidation, especially in the presence of oxygen and moisture, leading to the formation of carboxylic acids [45]. Furthermore, dialdehydes can readily undergo polymerization, forming complex oligomers or polymers, which can significantly alter their properties and applications [46]. Additionally, they could be used in the Schiff base formation reaction to produce novel materials with special properties, or they might be further oxidised to yield 2,3-dicarboxylic acid cellulose (DAC). Interestingly, the Schiff base DAC has developed into a hydrogel for drug-loading, opening the door for creative drug delivery methods [47]. Chitosan (Ch), recovered from seafood waste, is a biopolymer with vast potential for sustainable applications. Reactive NH_2_ groups on Ch easily “bond” with dialdehyde cellulose (DAC) through a Schiff base reaction. This creates new and exciting opportunities for the creation of composite or mixed materials, opening the door to a more sustainable future. Ch has a cycloaliphatic structure containing both active NH_2_ and OH groups. Ch can be crosslinked with polymers containing aldehydes despite its poor mechanical characteristics [48]. This study developed hydrogels by crosslinking dialdehyde cellulose (DAC) and chitosan (Ch). The resulting hydrogels, with their abundant chelation sites, amino, and hydroxyl groups, effectively encapsulate and protect pharmaceuticals. This intricate network ensures controlled release for enhanced therapeutic outcomes [48, 49]. Without the need for cross-linkers such as glutardehyde [50], a straightforward green preparation method for 4-AAP-embedded DAC/Ch hydrogels as drug loading carriers was developed using the Schiff base reaction. The generated DAC was evaluated by FTIR, XRD, and aldehyde content after being oxidised with sodium periodate using an environmentally safe microwave method. FT-IR, SEM/EDX, and 4-AAP release were used to examine the swelling characteristics and 4-AAP release of the resultant 4-AAP@DAC/Ch hydrogels. In addition, the hydrogel's chemical and physical properties were predicted using the DFT/B3LYP/6-31G (d) basis sets.

## Materials and methods

### Materials

The raw sugarcane bagasse (SC) used in this investigation was kindly donated by the Quena Company for Paper Industry located in Quena, Egypt. Ch and 4-AAP are purchased from Sigma-Aldrich. All of the materials and reagents used were used as it is.

### Preparation of α-cellulose

Starting with material labeled “SC,” a series of chemical treatments transformed it into a pulp high in alpha-cellulose. A 150 g sample of SC underwent a two-hour prehydrolysis in an autoclave, utilizing dilute hydrochloric acid at 120 °C. Following this, an alkaline treatment was performed. The prehydrolyzed product was combined with a sodium hydroxide solution (20 g NaOH in 300 ml water) and heated to 170 °C for two hrs, yielding a brownish, fibrous material. To purify the resulting pulp (80 g), a lignin removal step was executed. This involved bleaching with a 3% perchloric acid solution (2.4 g HClO_2_ in 4750 ml water) within an acetic acid environment at 80 °C for 2 h. The pH was maintained between 1 and 3 through the controlled addition of acetic acid, resulting in isolated α-cellulose [51].

### Estimation of α-cellulose, lignin, hemicellulose and ash content in SC

Lignin, α-cellulose, hemicelluloses, and ash content was estimated by Tappi standard method (T13 wd-74), (T203 OS-61), (T19 wd-71) as mentioned elsewhere [52].

For α-cellulose estimation, the cellulose was cut into thin pieces 3 × 3 mm then about 3 g (exactly weighed) were put into porcelain beaker (250 ml) then 25 ml of NaOH (17.5% wt/wt) were added, and after leaving to swell for 4 min (time exactly measured from the last drop), then the pulp was pressed with glass rode for 3 min. After pressing, another 25 ml of NaOH were added and mixed thoroughly till one gets a homogeneous paste (mixing for about 1 min). The beaker was then covered and left for 35 min, at 20 °C, then 100 ml of distilled water was added and quickly filtered under suction using a sintered glass funnel (II G2 of 5 cm, diameter and 4.5 in length), the filtrate was poured on the past twice before washing with distilled water. After washing with distilled water till neutrality, 100 ml of acetic acid of concentration 10% were added drop-wise for washing followed by distilled water. The temperature must be kept constant at 20 °C during the whole experiment. The α-cellulose was estimated gravimetrically after dryness in drying oven at 105 °C.

For lignin estimation, commonly known as Klason lignin. This method is essential for understanding the lignin content, which is a significant component of plant cell walls and affects the properties of wood and pulp products. 1 g are treated with sulfuric acid (72% wt/wt) to hydrolyze and solubilize the carbohydrates, leaving behind the acid-insoluble lignin for 2 h at room temperature. Then diluted to 3% sulfuric acid and boiled for 4 h. The lignin was filtered till neutrality, then gravimetrically estimated and ignited at 400 °C for 30 min and then at 850 °C for 45 min. The weight of the ash is then subtracted to give the exact lignin (%).

1 $$\text{Lignin }(\text{\%})=\frac{Weight \,of \,lignin - Weight \,of \,its \,ash}{Weight \,of \,dry \,sample} \times 100$$

Pentosan which constitutes the major part of hemicellulose fraction, upon treatment with dilute mineral acids they undergo hydrolysis to pentoses. These in turn are further reacted upon by the acid to give furfural. For hemicellulose estimation, 2 g weighed material was placed in the reaction vessel together with 75 ml of distilled water and some glass beads, 75 ml of hydrobromic acid were added to the reaction vessel and heating was started under controlled heating to obtain constant distillation rate, 50 ml of the distillate were collected in 15 min after which another 50 ml of 40% hydrobromic acid were added. The process was repeated 8 times and a total of 400 ml of the distillate obtained were transferred to a 500 ml measuring flask and completed to mark with distilled water. 50 ml of the latter solution were cooled to 12 °C and 2 ml of hydrochloric acid (13%), 10 ml ammonium molybdate (25 g/L) and 20 ml of 0.05 N bromide- bromate solution were successively added. After exactly 4 min of the appearance of the yellow color, about 10 ml of 10% potassium iodide solution were added, the mixture was allowed to stand for five minutes and the titration was carried out immediately against 0.05 N sodium thiosulphate solution using starch as indicator. The furfural was estimated according to the following equation:

2 $$\text{Pentosans }(\text{\%})=\frac{(ml \,of\, bromide\, bromate\, used) -(ml \,of \,thiosulfate\, used)}{Weight\, of \,dry \,sample} \times 3.3$$

This method is used to measure the ash left after the combustion of organic material. The ash was estimated by igniting in muffle furnace a weighed sample in a porcelain crucible for 30 min at 400 °C and for further 45 min at 850 °C. The percentage of ash was calculated from [53]:

3 $$\text{Ash }(\text{\%})=\frac{Weight\, of \,ash}{Weight\, of\, dry \,sample} \times 100$$

The final product contained an impressive 94.2% α-cellulose, with minimal lignin (0.3%), hemicellulose (3.4%), and ash (0.4%).

### Preparation of microwave assisted dialdehyde cellulose (DAC)

To increase the solubility of cellulose we have prepared DAC, 1.5 g of the resulting α-cellulose was suspended in 20 ml of water and oxidized with 2 g of sodium periodate. This suspension was treated in the microwave for 1–3 min at 700 W. The product, DAC, was then filtered, washed repeatedly with ethanol, and left to dry at room temperature overnight [47].

### Determination of aldehyde content in DAC

**Scheme 1:**
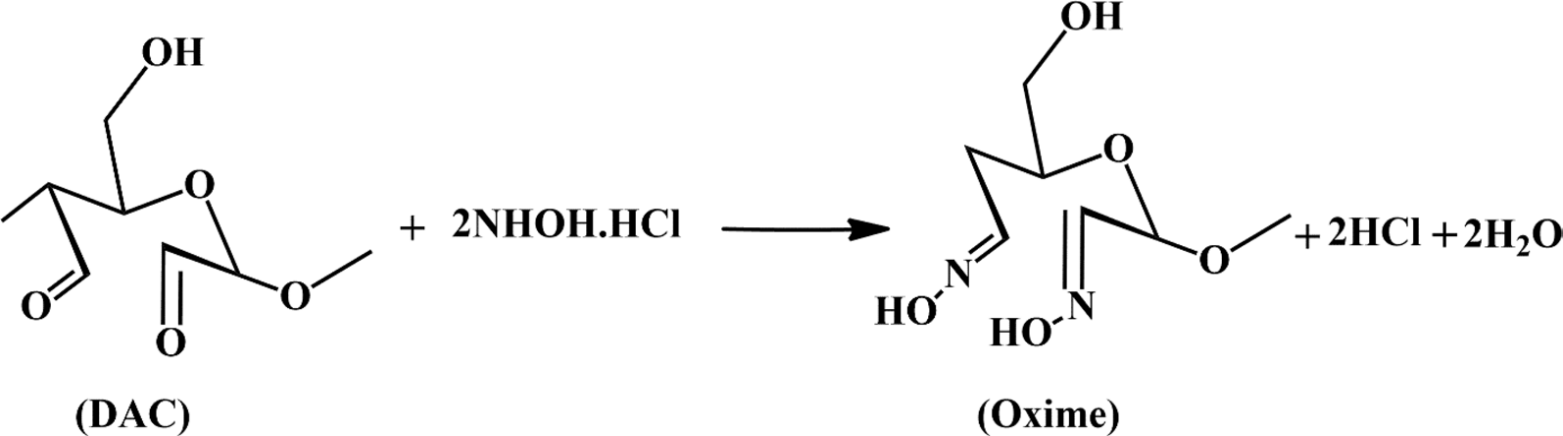
Schematic representation of the degree of oxidation measurement

To determine the degree of oxidation (D.O.) in DAC, we employed a reaction with hydroxyl amine hydrochloride (Scheme 1). Here, 0.1 g of DAC (undried) was added to a 250 ml beaker containing 25 ml of 0.25 M hydroxyl amine hydrochloride solution. The mixture was stirred with a magnetic stirrer for 48 h at room temperature, kept covered by aluminum foil. After filtration, the product was washed with 600 ml of deionized water and finally dried. As a control, the same concentration of cellulose solution (V_b_) was utilized. The released HCl was back-titrated with 1 M NaOH. The consumed NaOH solution was denoted as V_c_ (L) (scheme 1). Equation (4) was used to calculate the nitrogen concentration of the oxime derivative of DAC:

4 $$\text{DO }(\text{\%})=\frac{{(V}_{C-}{V}_{b}) X C}{8 X \frac{m}{M}} \times 100$$

where C = 1 M, m is the dry weight (m = 0.5 g) of DAC and M is approximately the molecular weight of the repeating unit in DAC [47].

### Preparation of dialdehyde cellulose/chitosan Schiff base hydrogels encapsulated with 4-aminoacetophenone drug

To create the DAC/Ch hydrogels, the chitosan solution (1 g/10 ml of water) was prepared by dispersing chitosan in acidic distilled water (diluted HCl solution with pH 4) heated to 40 °C. Then, they mixed this solution in equal parts with a similarly prepared DAC solution (1 g/10 ml of water). After thorough mixing for an hour, the combined solution was incubated at room temperature (37 °C) for 24 h, followed by a final cooling step at 4 °C for 4 h. This resulting hydrogel served as the control sample (DAC/Ch). Following the established procedure for the control DAC/Ch hydrogel, as detailed above, three additional hydrogels were prepared with the incorporation of 4-AAP. Specifically, 4-AAP was added at concentrations of 5%, and 7.5% (w/v) during the initial mixing stage of the Ch and DAC solutions. These samples are denoted as 4-AAP@DAC/Ch1 and 4-AAP@DAC/Ch2, respectively. It’s important to note that concentrations exceeding 7.5% 4-AAP did not result in increased drug loading, thus limiting our investigation to the aforementioned range.

### Characterizations

The diffraction patterns were obtained using a Bruker D-8 Advance X-ray diffractometer (Germany) at 40 kV and 40 mA with copper (K) radiation (1.5406).

5 $$\text{Cr}.\text{ I}=\frac{{I}_{t -}{I}_{\alpha }}{{I}_{t}}$$

where Cr.I is the crystallenity index, I_t_ is the total intensity of the crystalline peak for cellulose II at 21° and I_α_ is the amorphous intensity at 16° for cellulose II [54].

FTIR analysis was performed with a Mattson 5000 spectrometer (Unicam, United Kingdom) using the KBr disk method. The empirical crystallinity index (LOI) and mean hydrogen bond strength (MHBS) were calculated according to the following equations:

6 $$\text{LOI}=\frac{{A}_{1425}}{{A}_{900}}$$

7 $$\text{MHBS}=\frac{{A}_{OH}}{{A}_{CH}}$$

where A_1425_ and A_900_ refer to the FTIR absorbance at 1425 and 900 cm^−1^, respectively. In addition, A_OH_ and A_CH_ refer to the FTIR absorbance of the OH and CH peaks, respectively [10, 55].

The SEM, a Quanta-250 model, and the TEM, a JEOL JEM-2100 model, both used an electron beam with an energy of 120 kilovolts to achieve high-resolution images for detailed analysis.

The swelling of DAC/Ch and 4-AAP@DAC/Ch was described as following: a total of 0.1 g of each hydrogel was immersed in distilled water for 24 h. After eliminating excess water, the DAC/Ch, 4-AAP@DAC/Ch1 and 4-AAP@DAC/Ch1 hydrogels were weighed. The swelling ratios were calculated using the following Eq. (8):

8 $$\text{Swelling }\left(\text{\%}\right)=\frac{mt-m0}{m0} 100\text{\%}$$

where m_t_ and m_0_ are the mass of the swollen gel at time t and the mass of the dry hydrogel, respectively [13].

### Drug loading (${\mathbf{D}\mathbf{L}}_{4-\mathbf{A}\mathbf{A}\mathbf{P}}$)

The 4-AAP content in the DAC/Ch hydrogels was calculated according to the original concentration of 4-AAP and the concentration of 4-AAP in the solution after loading, which could be quantified using a UV‒Vis spectrophotometer at a wavelength of 316 nm. The 4-AAP loading efficiency (${DL}_{4-\text{AAP}}$) was calculated using Eq. (9):

9 $${DL}_{4-\text{AAP}} \left(\%, w/w\right)=\frac{\text{Weight of }4-\text{AAP in DAC}/\text{Ch }}{Initial \,weight \,of \,the \,DAC/Ch} \times 100$$

where weight of 4–AAP in DAC/Ch is (weight of drug loaded hydrogel—initial weight of the hydrogel) [56].

### 4-AAP release study (DR_4-AAP_)

The DR_4-AAP_ of 4-AAP from DAC/Ch was determined by using a dialysis membrane (12,000–14,000, AVWR Company). The 4-AAP@DAC/Ch hydrogels were placed in a buffer solution, and 5 ml of solution was collected at time intervals for determination of the drug content using a UV‒Vis spectrophotometer at 316 nm. An equal volume of the same solution medium was added back to maintain a constant volume. All the samples were subjected to triplicate in vitro release tests [57].

10 $${DR}_{4-\text{AAP}} \left(\%, w/w\right)=\frac{\text{Release of }4-\text{AAP at time }}{Total 4-\text{AAP drug} loaded \,on \,the\, 4-AAP@DAC/Ch } \times 100$$

### Release kinetics

The zero-order, first-order, Higuchi, Hixon-Crowell, and Korsmeyer-Peppas models were among the drug release models. To choose the model that fit the data the best, R^2^ values were employed.

11 $$\text{C }= {\text{K}}_{0}\text{t}$$

12 $$\text{log C }=\text{log C}0-\frac{K1t}{2.303}$$

13 $$\text{Q }= {\text{k}}_{\text{H}}{\text{t}}^{1/2}$$

14 $${\text{W}}_{0}-{\text{W}}_{\text{t}} = {\text{k}}_{\text{HC}}\text{t}$$

15 $$\text{log }C=\text{log kkp }+\text{ nlog t}$$

where k_0_, k_1_ k_H_, k_HC_ and k_KP_ are the zero, first-order, Higuchi, Hixon–Crowell and Korsmeyer–Peppas rate constants, respectively. C is the cumulative 4-AAP DR, t is the time, C_0_ is the initial concentration of 4-AAP, K_1_ is the first-order constant, and n is the release exponent [3, 58].

### Computational procedures

The Gaussian 09 program was used to simulate the molecules using a method called density functional theory (DFT). Specifically, they employed a type of DFT called B3LYP along with a specific set of mathematical functions (basis set) to describe the electron distribution. The calculations aimed to determine various properties of the molecules, including their optimal structures, energies, response to electric fields, and how easily they gain or lose electrons. Where the total energy (E_T_), the energy of the highest occupied MO E_HOMO_, the energy of the lowest unoccupied MO E_LUMO_, the energy gap (E_g_), the dipole moment (μ), the absolute electronegativity (χ), the chemical potential (Pi), the absolute hardness (η), the absolute softness (σ), the global electrophilicity (ω), the chemical softness (S), and the additional electronic charge (ΔN_max_) were calculated as following [32, 59, 60]:

16 $${E}_{gap}=({E}_{LUMO}-{E}_{HOMO})$$

17 $$\upchi =\frac{- ({E}_{HOMO}+ {E}_{LUMO}) }{2}$$

18 $$\text{Pi}=-\upchi$$

19 $$\upeta =\frac{({E}_{LUMO}+ {E}_{HOMO}) }{2}$$

20 $$\upsigma =\frac{1 }{\upeta }$$

21 $$\text{S}=\frac{1 }{2\upeta }$$

22 $$\Delta {N}_{max}=\frac{-\text{Pi }}{\upeta }$$

23 $$\upomega =\frac{{ Pi}^{2} }{2\upeta }$$

Adsorption energy (${E}_{Ads}$) was computed using an equation to corroborate the interaction of the 4-AAP medication with the functionalized DAC/Ch hydrogel.

24 $${E}_{Ads}=({E}_{4-AAP@DAC/Ch}-{(E}_{DAC/Ch}+{E}_{4-AAP}))$$

Here, E is the total energy [61].

## Results and discussion

### Dialdehyde cellulose/chitosan schiff base hydrogel mechanism

**Scheme 2:**
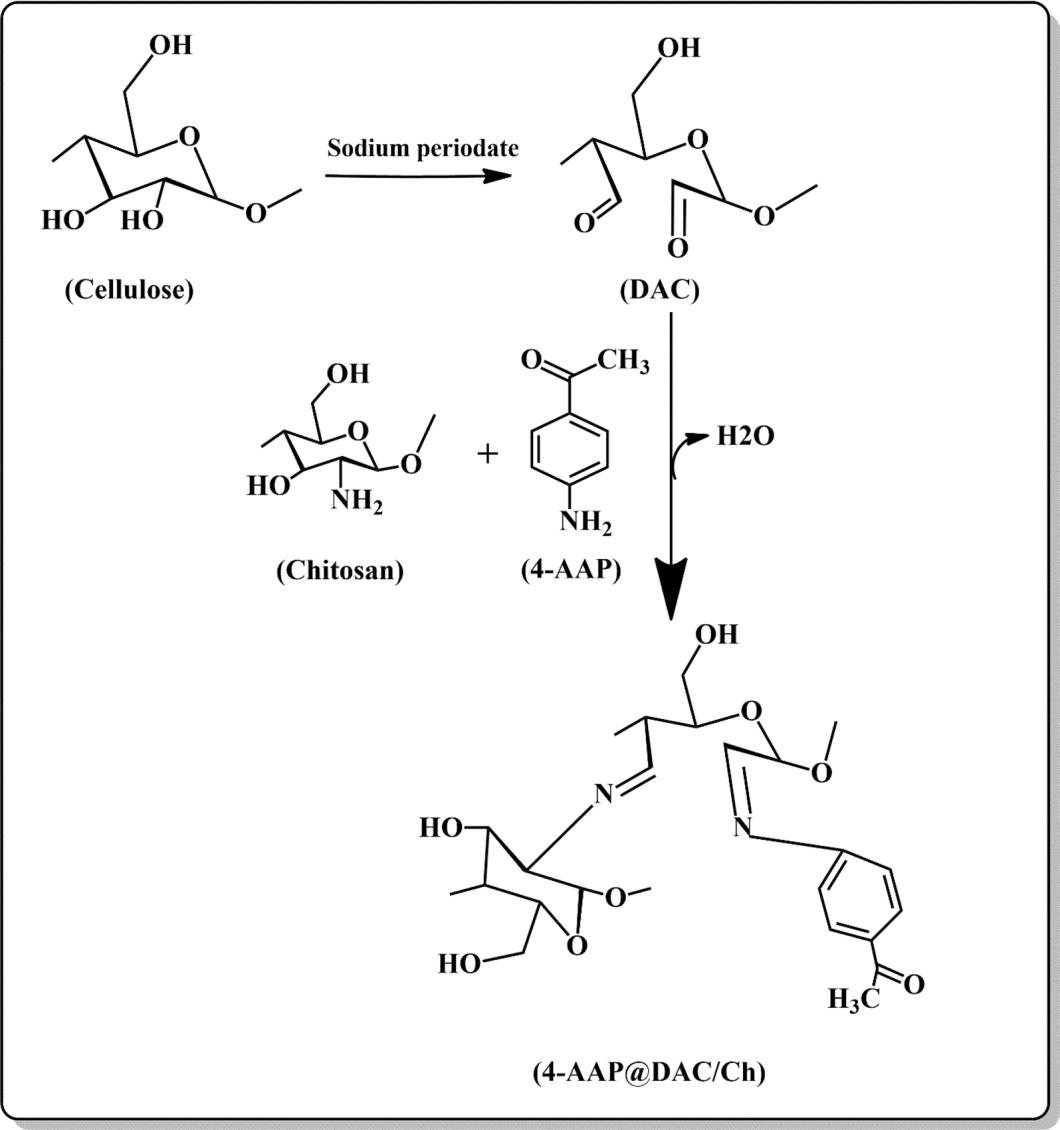
Reaction mechanism for the preparation of the 4-AAP@DAC/Ch hydrogel

The D.O. of the DAC was 28.57%. NaIO_4_ was used to break down the cellulose ring and prepare two free aldehyde groups at C_2_ and C_3_. The C = O of DAC reacts with the NH_2_ groups of both chitosan and 4-AAP by imine bond formation (C = N) via Schiff bases, as shown in Scheme 2.

### Computational procedures

**Fig. 1:**
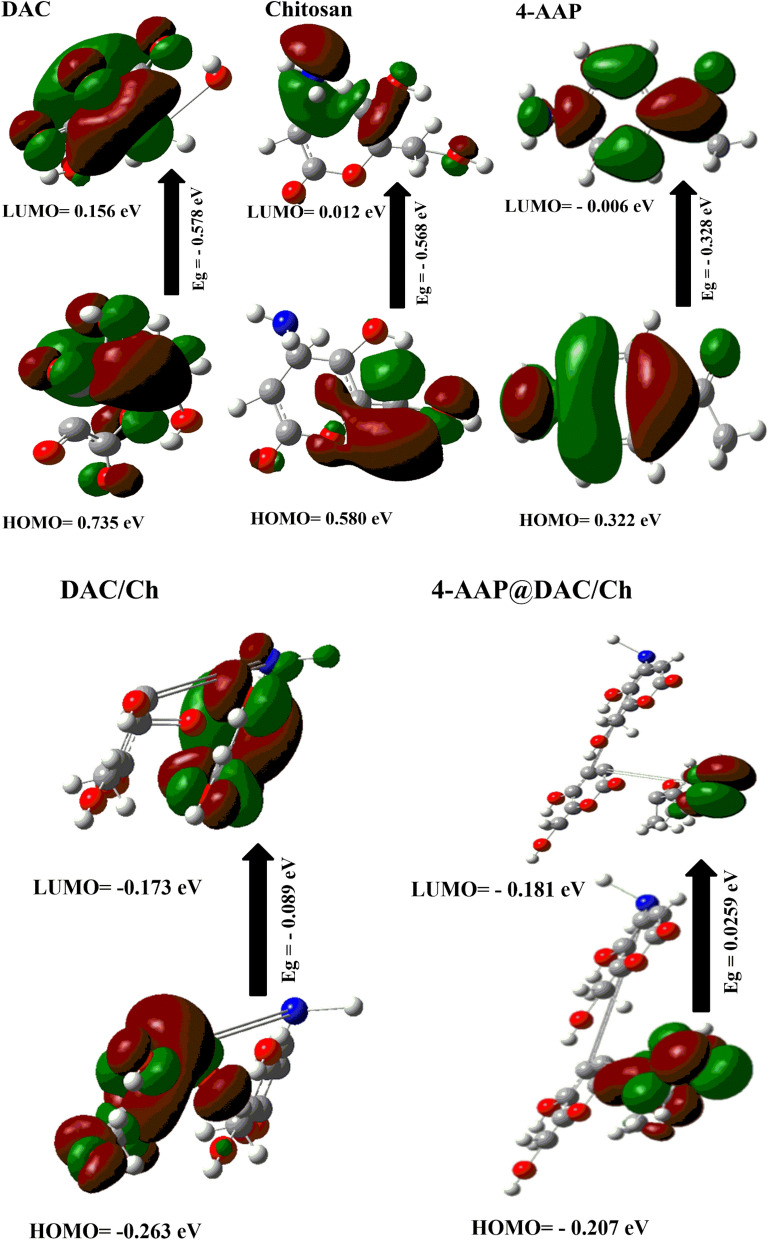
The gap energies (HOMO–LUMO) (eV) were calculated for the hydrogels using DFT B3LYP/6-31G (d), as was the molecular orbital interaction between DAC, 4-AAP and chitosan

DFT calculations were employed to study the stability of the DAC, chitosan, 4-AAP, DAC/Ch and 4-AAP@DAC/Ch hydrogels. From Fig. 1 and Table 1 reveals that the 4-AAP@DAC/Ch formulation exhibits distinct properties compared to its individual components and DAC/Ch: it is significantly softer, indicated by a lower energy value (38.610 eV), and displays a higher ω value (1.464 eV), suggesting strong energy changes between HOMO and LUMO [62]. The negative Pi values for 4-AAP@DAC/Ch particularly the more negative value for the latter (– 0.373), confirm the 4-AAP@DAC/Ch stability [55]. The reduced E_g_ in 4-AAP@DAC/Ch (0.086 eV) demonstrates enhanced chemical reactivity and charge transfer, signifying a strong chemical interaction between DAC, chitosan, and 4-AAP, and highlighting the influence of the electron-acceptor group on LUMO stabilization and biological activity. Additionally, the small energy gap indicated that the hydrogel was more reactive due to the ease of electron transfer from the HOMO to the LUMO. The hydrogel was formed through interactions between DAC and chitosan without/with 4-AAP (i.e., DAC/Ch and 4-AAP@DAC/Ch), and the E_g_ decreased for 4-AAP@DAC/Ch (i.e., 0.086 eV), demonstrating that charge transfer occurs much more within 4-AAP@DAC/Ch, which in turn proves the strong chemical reaction between DAC, chitosan and 4-AAP. Consequently, the lowering of the HOMO–LUMO energy gap is essentially a consequence of the large stabilization of the LUMO due to the strong electron-accepting capability of the electron-acceptor group and influences the biological activity of the molecule (Fig. 1) [55, 60]. As shown in Table 1, Pi is negative for DAC/Ch and 4-AAP@DAC/Ch (i.e., − 0.218 and − 0.373), which means that DAC/Ch and 4-AAP@DAC/Ch are stable [63]. The E_T_ (− 1254.57 au) and μ (6.341 Debye) of 4-AAP@DAC/Ch represent its polarity. The E_ads_ for 4-AAP@DAC/Ch (− 73.342 au) is high, indicating slow 4-AAP release, which is in accordance with experimentally observed results [61].

**Table 1:** The quantum chemical parameters of DAC, chitosan, 4-AAP, DAC/Ch and the 4-AAP@DAC/Ch hydrogel

DFT B3LYP/6-31G (d)	DAC	Chitosan	4-AAP	DAC/Ch	4-AAP@DAC/Ch
E_LUMO_ (eV)	0.156	0.012	− 0.006	− 0.173	− 0.181
E_HOMO_ (eV)	0.735	0.580	0.322	− 0.263	− 0.207
E_T_ (au)	0.360	1.431	− 0.028	− 1181.2	− 1254.57
E_g_ (eV)	− 0.578	− 0.568	− 0.328	0.089	0.025
ɳ (eV)	− 0.289	− 0.284	− 0.164	0.044	0.012
χ (eV)	− 0.445	− 0.296	− 0.158	0.218	0.194
Pi (eV)	0.445	0.296	0.158	− 0.218	− 0.194
σ (eV)	− 3.454	− 3.518	− 6.079	22.376	77.22
S (eV)	− 1.727	− 1.759	− 3.039	11.188	38.610
ω (eV)	− 0.343	− 0.154	− 0.075	0.534	1.464
ΔN max	− 1.540	− 1.043	− 0.96079	− 4.889	− 15.038

### XRD study

**Fig. 2:**
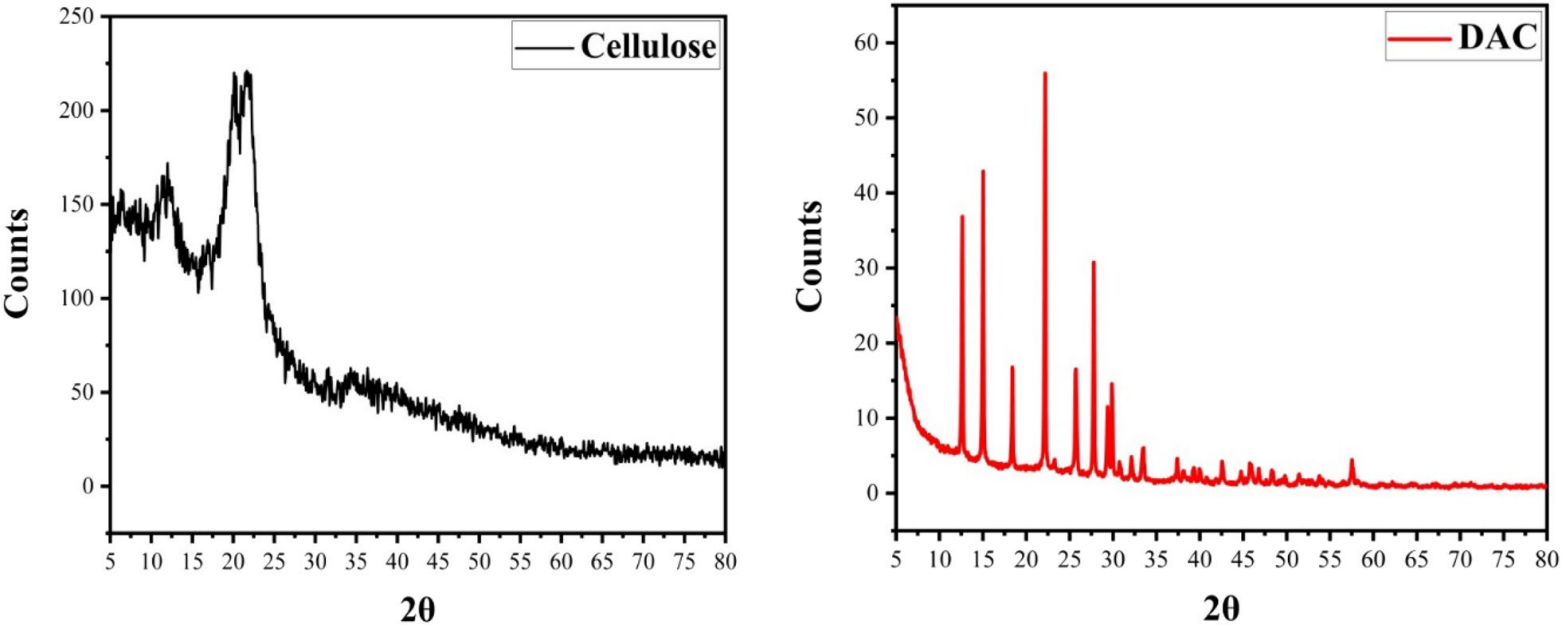
XRD spectra of cellulose and DAC

Like those of usual cellulose II peaks, the peak positions at 2 = 12, 20, and 21° were assigned to diffraction planes (110), (110), and (020), respectively [12, 54]. The X-ray diffraction patterns of DAC revealed peaks with high intensity which caused due to NaIO_3_, prove its high crystallinity. Despite multiple washes of the DAC product, oxidant (-IO_3_) diffraction signals were still discernible in the diffraction patterns. The results showed that DAC formed a stable combination with a reduced form of oxidant (-IO_3_), indicating that it is crystalline [47]. The cellulose chains were completely degraded, resulting in a decrease in Cr.I from 43% in cellulose II to 34% in DAC and structural alterations after oxidation. In the DAC, the broad peak at (2θ) 21°, which corresponds to the cellulose crystallinity area, is diminished (Fig. 2). The loss of Cr.I is caused by the ring-opening production of glucose units and the dissolution of their structure [47, 64].

### FTIR spectra

**Fig. 3:**
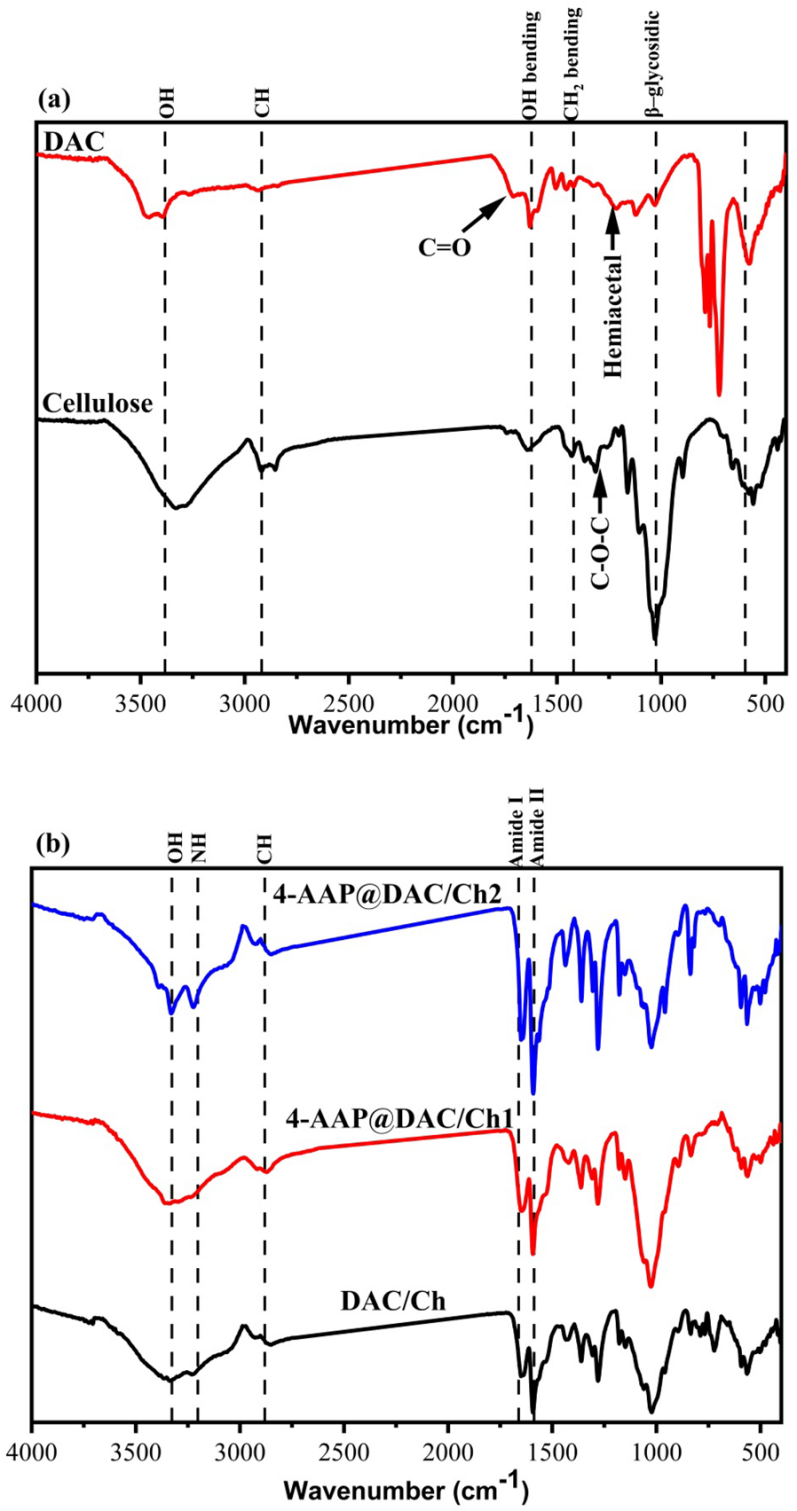
FTIR of (**a**) cellulose and DAC and (**b**) DAC/Ch, 4-AAP@DAC/Ch1 and 4 AAP@DAC/Ch2

Cellulose showed characteristic bands at 3462 (O–H), 2882 (C–H), 1650 (OH bending of the adsorbed water), 1375 (CH_2_ bending and C–O–C of pyranose ring vibration) and 1040 cm^−1^ (β–glycosidic linkage between glucose units in cellulose) [12, 51]. In addition to the main characteristic bands of cellulose, DAC contained new characteristic absorption bands at 1716 and 1224 cm^−1^ corresponding to the C = O of the aldehyde group and hemiacetal bonds between the aldehyde groups and their neighboring OH groups, respectively (Fig. 3) [47, 65]. It was feasible to produce a DAC/Ch hydrogel by forming a Schiff base between the amino groups in Ch and the aldehyde groups in DAC. Aldehyde can interact dynamically with various ketones that include amino groups, such as primary amines, hydroxylamines, and hydrazine, to make a Schiff base, which results in an imine bond linkage (C = N) [47].

FTIR analysis supported the occurrence of imine bonds in the hybrid gels. The broad band at 3330–3336 cm^−1^ in the FTIR spectra of the DAC/Ch hydrogels corresponds to OH vibrations. Amides I and II are assigned to the bands at 1647–1654 cm^−1^ and 1591–1593 cm^−1^, respectively [60, 66]. Further confirmation of the reaction between the aldehyde groups of DAC and the amino groups of Ch was the absence of the aldehyde peak at 1716 cm^−1^, which must be found in the FTIR spectrum of DAC. Likewise, it was expected that the peak for the amide I group would overlap with the peak for imine bond formation C = N (the Schiff base) in the DAC/Ch spectrum, which is localized at 1631 cm^−1^ [47].

From FTIR observations, we found that the O–H group of DAC/Ch was shifted from a high value (3336 cm^−1^) to a lower value (3330 cm^−1)^ for 4-AAP@DAC/Ch2, indicating strong H–bonding between DAC/Ch and 4-AAP (Fig. 3) [33, 67]. The calculated MHBS and LOI for 4-AAP@DAC/Ch2 are the highest due to the strong H–bonding between 4-AAP and DAC/Ch (Table 2).

**Table 2:** MHBS of DAC/Ch, 4-4 AAP-DAC/Ch1 and 4-4 AAP-DAC/Ch2

Sample	MHBS (A_OH_/A_CH_)	LOI (A_1425_/A_900_)
DAC/Ch	0.95	1.05
4-AAP@DAC/Ch1	0.96	1.06
4-AAP@DAC/Ch2	0.97	1.10

### Swelling study

**Fig. 4:**
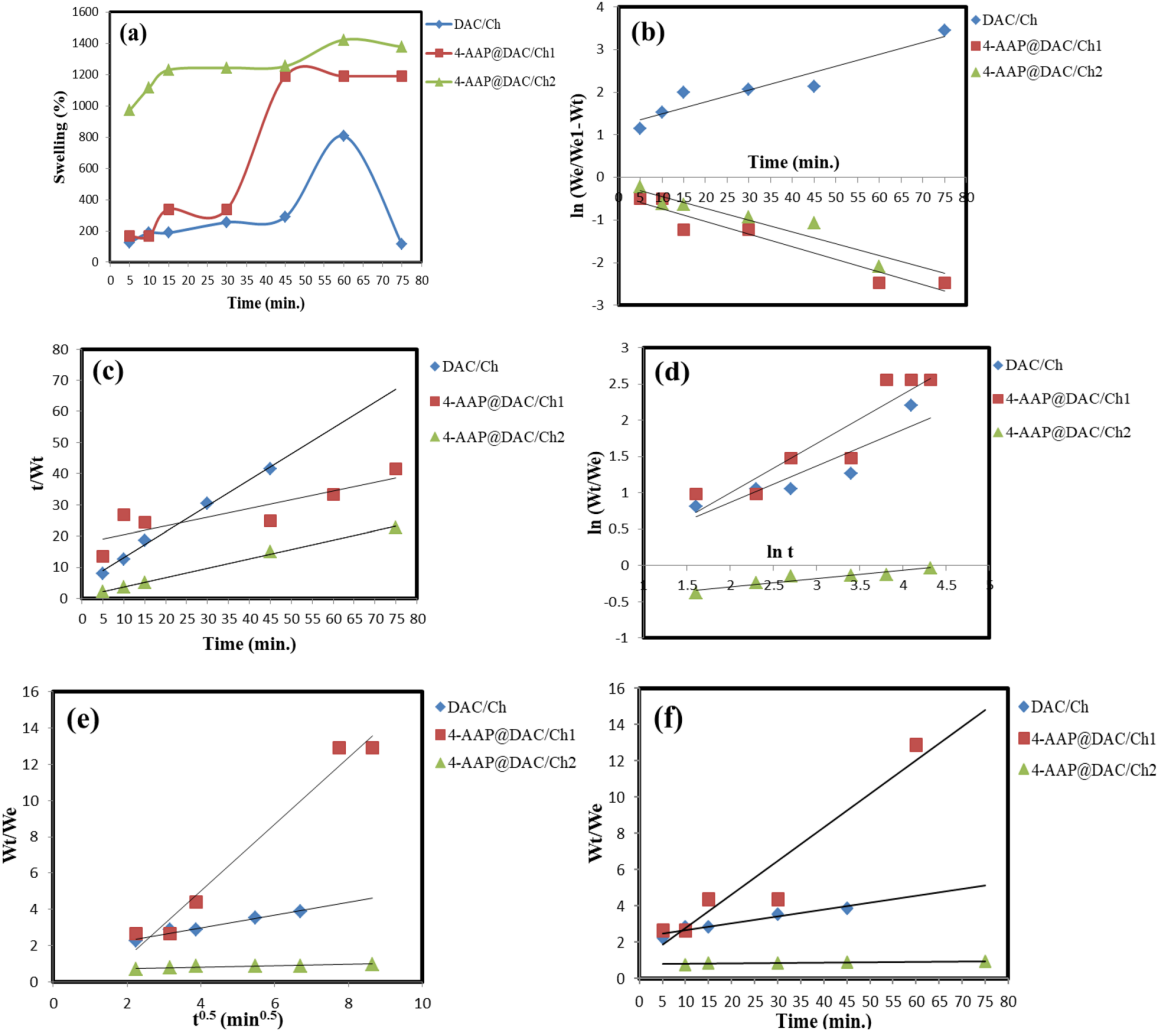
**a** Swelling behavior, **b** pseudo-first-order, **c** pseudo-second-order, **d** Peppas and Peppas, **e** Fick’s mechanism, and **f** Peppas and Franson method of DAC/Ch, 4-AAP@DAC/Ch1 and 4-AAP@DAC/Ch2

The swelling ratio increases quickly at first and then slowly until it reaches maximum continuous swelling. This is an important feature that may be noted in the swelling ratio vs. time curves. The swelling equilibrium (sw_eq._%) of the DAC/Ch, 4-AAP@DAC/Ch1 and 4-AAP@DAC/Ch2 gels was measured at 75 min, at pH 7.0, at 808.21, 1172.61 and 1422.61%, respectively (Fig. 4a). 4-AAP and DAC are highly hydrophilic and anionic due to the presence of amino and aldehyde groups. With increasing 4-AAP content in the hydrogel, the amount of sw_eq._% in the 4-AAP@DAC/Ch2 gels increased. The swelling of -AAP@DAC/Ch2 was greater than the swelling of DAC/Ch and 4-AAP@DAC/Ch1 because of the hydrophilicity of 4-AAP. Due to the numerous highly charged groups of 4-AAP and DAC, the structure of 4-AAP@DAC/Ch2 has strong electrostatic interactions. The positively charged groups produced by nitrogen in the amino groups of 4-AAP increase the electrostatic double layer attraction to the negatively charged DAC. Sondari et al. successfully synthesized a DAC/Ch hydrogel from sugarcane waste leaves, demonstrating moderate swelling (120–450%) [68]. However, our study significantly surpasses these swelling capabilities, achieving values of 808.21%, 1172.61%, and 1422.61%, indicating a potentially more porous and hydrophilic structure.

When comparing the two kinetic models (pseudo-first and pseudo-second orders), we found that K_2_ provided the best R^2^ (0.9090) and W_e2_ (891.82%) values for DAC/Ch. Therefore, chemisorption processes (i.e., related to pseudo-second-order processes) were observed for DAC/Ch. In contrast, for 4-AAP@DAC/Ch1, the best R^2^ (0.9283) was provided by K_1_, but W_e2_ (1927.86%) was close to W_e;exp_ (1172.61%) (i.e., related to pseudo-first-order and second-order reactions). 4-AAP@DAC/Ch1 exhibited both physisorption and chemisorption. For 4-AAP@DAC/Ch2, the best R^2^ (0.9977) was provided by K_2_, and W_e2_ (1500.76%) was close to W_e;exp_ (1422.61%) (i.e., related to pseudo-second-order). The results showed that 4-AAP@DAC/Ch2 undergo chemisorption (Fig. 4c) [10].

For DAC/Ch, 4-AAP@DAC/Ch1; n = 0.683 and 0.506; indicates non-Fickian diffusion (Fig. 4d). The D value of 4-AAP@DAC/Ch2 improved with increasing 4-AAP content in the networks (i.e., 0.121) due to its increased swelling capacity (Fig. 4e) [7, 10]. The values of the diffusion coefficient D for the prepared 4-AAP@DAC/Ch2 improved with increasing 4-AAP content due to the resulting higher swelling capacity. 4-AAP@DAC/Ch2 had the highest υ value (24.55), indicating that it had a greater swelling capacity than did DAC/Ch and 4-AAP@DAC/Ch1 (Table 3, Fig. 4f).

**Table 3:** Experimental swelling (We; exp. (g/g)) and model swelling % We 1; calc. (g/g); We 2; calc. (g/g) HPMC@AM-co-SPA hydrogels

Kinetic model	Parameter	Hydrogels		
		DAC/Ch	4-AAP@DAC/Ch1	4-AAP@DAC/Ch2
Pseudo first order	W_e; exp._ (%)	808.21	1172.61	1422.61
	W_e 1; calc._ (%)	205.72	446.84	1509.44
	K_1_	0.007	0.031	0.028
	R^2^	0.8835	0.9283	0.8965
Pseudo second order	W_e 2; calc._ (%)	891.82	1927.86	1500.76
	K_2_	0.194	0.018	0.009
	R^2^	0.9090	0.7927	0.9977
Peppas and Peppas	K_3_	0.149	0.371	0.537
	n	0.683	0.506	0.121
	R^2^	0.8093	0.8591	0.8867
Fick’s mechanism	D	0.0036	0.351	1.93
	R^2^	0.9672	0.9313	0.9083
Peppas and Franson method	$\upsilon$	0.0378	0.184	24.55
	R^2^	0.9283	0.9147	0.8378

### Drug loading and drug release study

**Fig. 5:**
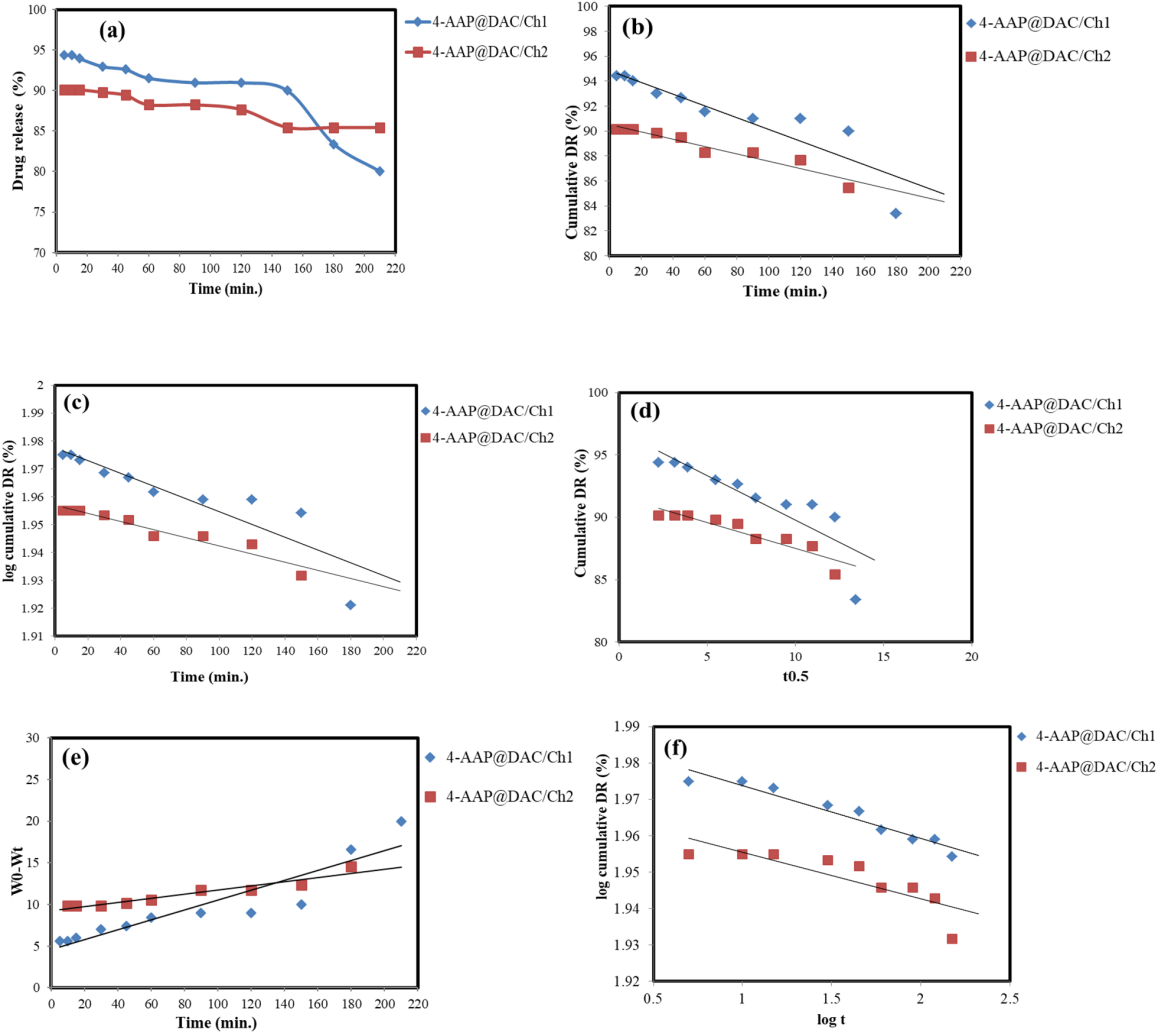
Kinetic models for 4-AAP release from 4-AAP@DAC/Ch1 and 4-AAP@DAC/Ch2 (**a**) Zero-order, **b** pseudo-first-order, **c** Higuchi model, (**d**) Hixson-crowell model, and **e** Korsmeyer-Peppas model

After releasing of 4-AAP and removing of 4-AAP@DAC/Ch1 and 4-AAP@DAC/Ch2, the amount of loaded 4-AAP was proportional to its concentration and could be determined at 316 nm. The ${DL}_{4-\text{AAP}}$ calculated values were 94.40 and 90.14%, respectively. This means that while more 4-AAP was initially present in the 4-AAP@DAC/Ch2 preparation, the hydrogel structure was unable to efficiently incorporate and retain the excess drug. The 5% concentration, in contrast, likely provided an optimal balance, allowing for efficient drug encapsulation without overwhelming the hydrogel’s capacity. Furthermore, the fact that concentrations exceeding 7.5% did not yield increased loading reinforces the idea of a saturation limit within the hydrogel matrix. The 4-AAP@DAC/Ch formulation was able to slow the release of the 4-AAP drug. Figure 5a shows the release profiles of 4-AAP from 4-AAP@DAC/Ch1 and 4-AAP@DAC/Ch2. The observed drug release profiles for 4-AAP@DAC/Ch1 and 4-AAP@DAC/Ch2, reaching 14.57% and 20% respectively within the first 210 min. The relatively low continued release rate suggests several potential limiting factors. Firstly, the initial burst likely corresponds to 4-AAP molecules situated near the hydrogel’s surface, readily diffusing into the release medium. As these superficial molecules are depleted, subsequent release relies on diffusion from deeper within the hydrogel matrix. The structure of the DAC/chitosan network, with its potential for limited tortuous pathways, could impose significant diffusion barriers, slowing down the release process. Secondly, strong interactions, such as electrostatic or hydrogen bonding, between the 4-AAP molecules and the hydrogel matrix may hinder their release. A portion of the drug may be tightly bound, necessitating more time or energy to detach. Thirdly, the hydrogel's degradation rate could be a limiting factor. If the release mechanism is primarily diffusion-driven rather than degradation-mediated, the remaining drug will be retained within the matrix until other factors facilitate its release. Fourthly, an uneven drug distribution, with most of the drug on the surface, would lead to a rapid initial release, followed by a slow release of the deeply embedded 4-AAP drug. Finally, it is possible that the matrix has reached a saturation point, where the remaining drug is very difficult to extract, requiring a longer period or different release conditions to achieve a more complete release. Therefore, further studies are needed to optimize the hydrogel’s structure, drug loading, and release conditions to achieve a more sustained and complete 4-AAP drug delivery profile.

The R^2^ comparison revealed that the highest R^2^ value for 4-AAP@DAC/Ch1 was obtained for the Hixson–Crowell model (0.9187), which showed that the 4-AAP drug is released from 4-AAP@DAC/Ch1 where there is a change in diameter [69]. For 4-AAP@DAC/Ch2, the zero model had a greater R^2^ (i.e., 0.9561) than did the other models. Hence, the 4-AAP release profile of 4-AAP@DAC/Ch2 is independent of the amount of 4-AAP [69, 70]. Finally, the Korsmeyer–Peppas power law equation states that n is 0.014 and 0.013 (i.e., less than 0.5) for 4-AAP@DAC/Ch1 and 4-AAP@DAC/Ch2, respectively, which implies Fickian diffusion (Table 4, Fig. 5) [69].

**Table 4:** Kinetic models for 4-AAP release from 4-AAP@DAC/Ch1 and 4-AAP@DAC/Ch2

Kinetic model	Parameter	4-AAP@DAC/Ch1	4-AAP@DAC/Ch2
Zero order	K_0_	28 × 10^–3^	22 × 10^–3^
	R^2^	0.7952	0.9561
First order	K_1_	77 × 10^–4^	95 × 10^–6^
	R^2^	0.7857	0.9536
Higuchi model	K_H_	32 × 10^–2^	36 × 10^–2^
	R^2^	0.9090	0.9003
Hixson-crowell model	K_HC_	− 368.46	38.38
	R^2^	0.9187	0.8379
Korsmeyer-Peppas model	N	0.014	0.013
	k_KP_	2.62	35 × 10^–4^
	R^2^	0.8521	0.9064

### SEM analysis

**Fig. 6:**
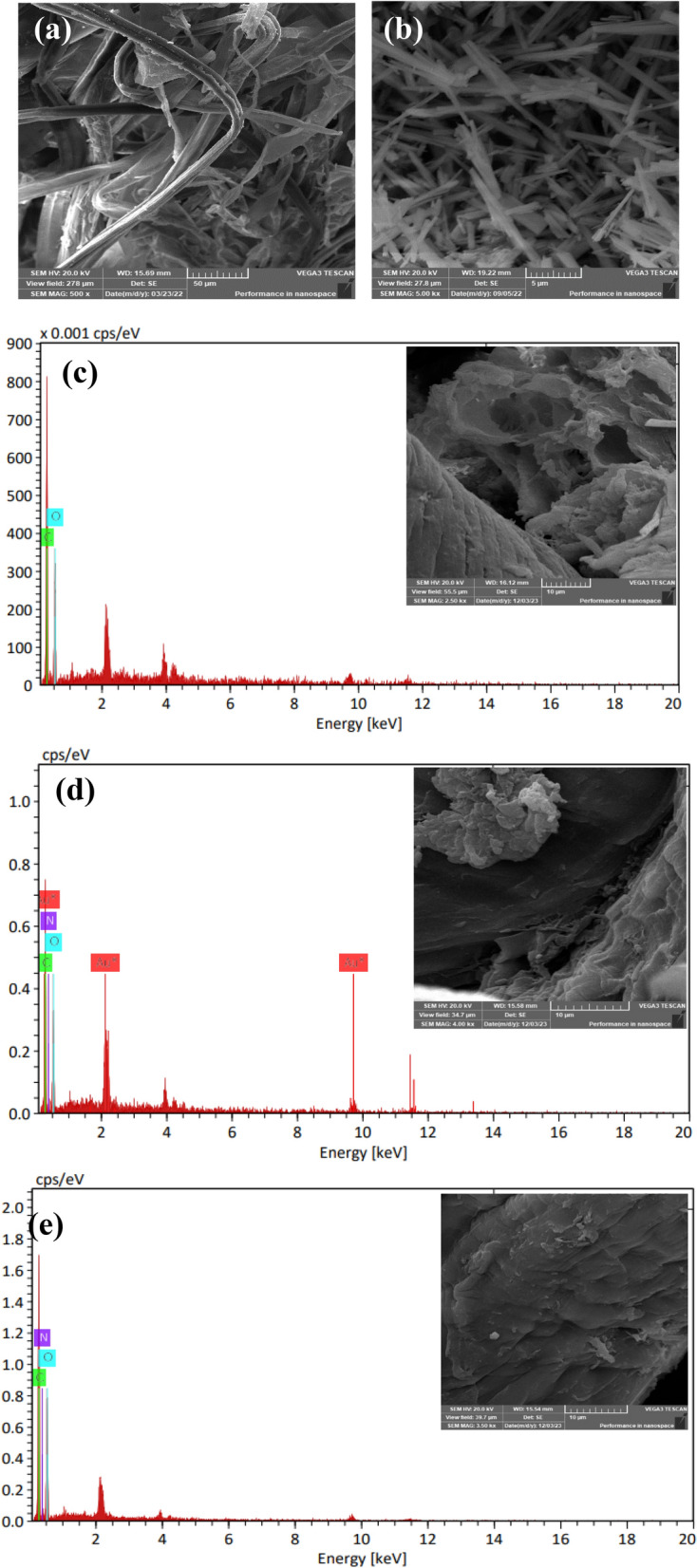
SEM analysis of (**a**) cellulose, (b) DAC and SEM/EDX analysis for (**c**) DAC/Ch, **d** 4-AAP@DAC/Ch1 and (**d**) 4-AAP@DAC/Ch2

The cellulose appeared as a long rod that broke after oxidation to small rods with a length ≈ 7.92 nm for DAC (Fig. 6a, b). SEM analysis of the DAC/Ch, 4-AAP@DAC/Ch1 and 4-AAP@DAC/Ch2 hydrogels revealed the surface morphology and how the presence of 4-AAP affected the appearance of the DAC/Ch surface. DAC/Ch exhibited numerous pores (size ≈ 5.52 nm), while the pores on 4-AAP-DAC/Ch1 and 4-AAP-DAC/Ch2 disappeared due to the encapsulation of 4-AAP (Fig. 6c, d, e). EDX analysis of DAC/Ch (C ≈55.40% and O ≈ 4.60%), 4-AAP@DAC/Ch1 (C ≈53.75%, O ≈ 37.85% and N ≈6.47%) and 4-AAP@DAC/Ch2 (C ≈55.68%, O ≈ 39.17% and N ≈7.23%) revealed the percentage of atoms (Fig. 6c, d, e).

## Conclusions

This study not only focused on the development of drug delivery system but also emphasized sustainable resource utilization. Dialdehyde cellulose (DAC), a key component of our hydrogel matrix, was derived from recycled sugarcane bagasse (SC), a readily available agricultural waste product. By repurposing this biomass, we aimed to demonstrate the feasibility of creating high-value biomaterials while minimizing environmental impact. Our objective was to explore the potential of recycled DAC in conjunction with chitosan to fabricate hydrogels capable of controlled 4-aminoantipyrine (4-AAP) release, thereby contributing to both pharmaceutical advancements and circular economy principles. This DAC was reacted with chitosan and 4-AAP via the Schiff base method by producing imine bonds between the C = O orbital of DAC and the NH_2_ orbital of chitosan and 4-AAP. This chemical reaction was proven by FTIR spectroscopy, where the amide I group overlapped with the peak corresponding to imine bond formation (C = N; the Schiff base bond) in the DAC/Ch spectrum, which was localized at 1631 cm^−1^. The prepared 4-AAP@DAC/Ch1 and 4-AAP@DAC/Ch2 had DL% values of 5.60 and 9.86%, respectively. The 4-AAP@DAC/Ch formulation was able to slow the release of the 4-AAP drug. This was proven by the release study and DFT. Sw% of 4-AAP@DAC/Ch2 (1172.61%) ˃ 4-AAP@DAC/Ch1 (808.21%). This difference may be due to the strong interactions and high number of imine bonds that formed with the high content of 4-AAP. SEM analysis revealed that the pore size decreased with increasing 4-AAP content. The 4-AAP is a soluble polymer. Therefore, the 4-AAP@DAC/Ch matrix manufactured in this research paper exhibited an extended release profile and delayed solubility. While 4-AAP@DAC/Ch2 initially exhibited higher drug loading, its release efficiency was lower compared to 4-AAP@DAC/Ch1, suggesting a potential saturation effect and less effective drug retention. The release profiles of both hydrogels showed an initial burst followed by a slow, sustained release, likely due to a combination of surface drug diffusion, matrix diffusion barriers, strong drug-matrix interactions, and potentially uneven drug distribution. Kinetic modeling revealed that 4-AAP release from 4-AAP@DAC/Ch1 followed a Hixson-Crowell model, indicating a change in diameter during release, while 4-AAP@DAC/Ch2 followed a zero-order model, signifying release independent of drug amount. Furthermore, both hydrogels exhibited Fickian diffusion, as indicated by the Korsmeyer-Peppas model. These findings highlight the need for further optimization of hydrogel structure, drug loading, and release conditions to achieve a more controlled and complete 4-AAP delivery profile. All of the above chemical aparameters, such as high E_ads_ and ω, negative Pi, and low E_g_, indicate that 4-AAP@DAC/Ch can be developed as an efficient drug vehicle formulation.

## Data Availability

The datasets used and/or analysed during the current study are available from the corresponding author on reasonable request.
